# EFFICACY OF MALNUTRITION SCREENING AMONG OLDER VETERANS AT AN URBAN VA HOSPITAL

**DOI:** 10.1093/geroni/igad104.3294

**Published:** 2023-12-21

**Authors:** Sarah Cassatt, Elizabeth Parker

**Affiliations:** UMBC, Baltimore, Maryland, United States; UMSOM, Baltimore, Maryland, United States

## Abstract

Nearly half of the Veteran population is >65 years. Older Veterans are at increased risk of malnutrition associated with increased mortality, morbidity, length of stay, and readmissions. Malnutrition-focused quality measures are needed to combat this public health issue. The purpose of this study was to identify gaps in malnutrition screening at an urban VA facility. Chart auditing was conducted for all medical admissions during 72 hour periods across 14 months. Auditing determined if nutrition screening was completed, with an admission weight, and if nutrition was consulted for nutritionally compromised Veterans. Screening was conducted using the Malnutrition Screening Tool (MST). Additionally, estimated prevalence of nutritionally compromised Veterans was assessed using auditing data from 30 days. Audit data showed that on average only 57.4% (n=458) of Veterans admitted for inpatient care were screened correctly. Of those screened incorrectly, the three most common errors were 1) not collecting a weight on admission (N=82 (42.1%)); 2) inaccurate weight change interpretations (N=41 (21%)); 3) failure to refer nutritionally at-risk Veterans to nutrition services (N=38 19.5%)). Prevalence auditing demonstrated over 1/3 of Veterans were nutritionally compromised at the start of their hospitalization (36.43%, (N=295)). With an increasingly aging population in the United States there is a focus on shifting to value based payment systems. The new CMS Global Composite Malnutrition Score will include evaluation of hospitals malnutrition screening processes. There is a significant opportunity to close these gap areas and to adopt clinically relevant best practices to provide nutrition care earlier to at risk Veterans.

